# Supplementary material for: Unveiling the gut bacteriome diversity and distribution in the national fish hilsa (Tenualosa ilisha) of Bangladesh
Source: PLoS One. 2024 May 1;19(5):e0303047. doi: 10.1371/journal.pone.0303047 (PMC11062526; doi:10.1371/journal.pone.0303047)
Supplement: S1 File — (DOCX) [file pone.0303047.s003.docx]

**Supplementary Figures and Tables**


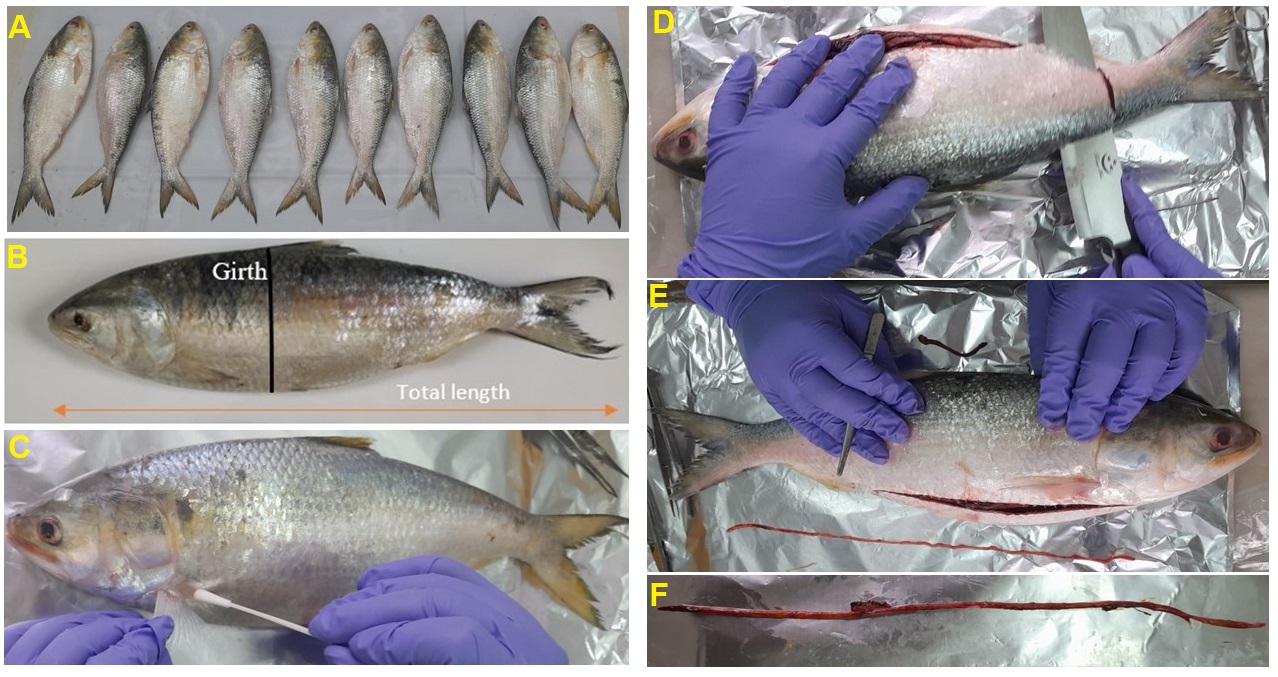


**Fig. S1.** Collected hilsa fishes used in this study. (A). Some of the collected hilsa fishes, (B) measuring length and girth, (C) screening for any injuries or skin lesions, (D-E) dissection for gut sample collection, and (F) gut of a hilsa fish.


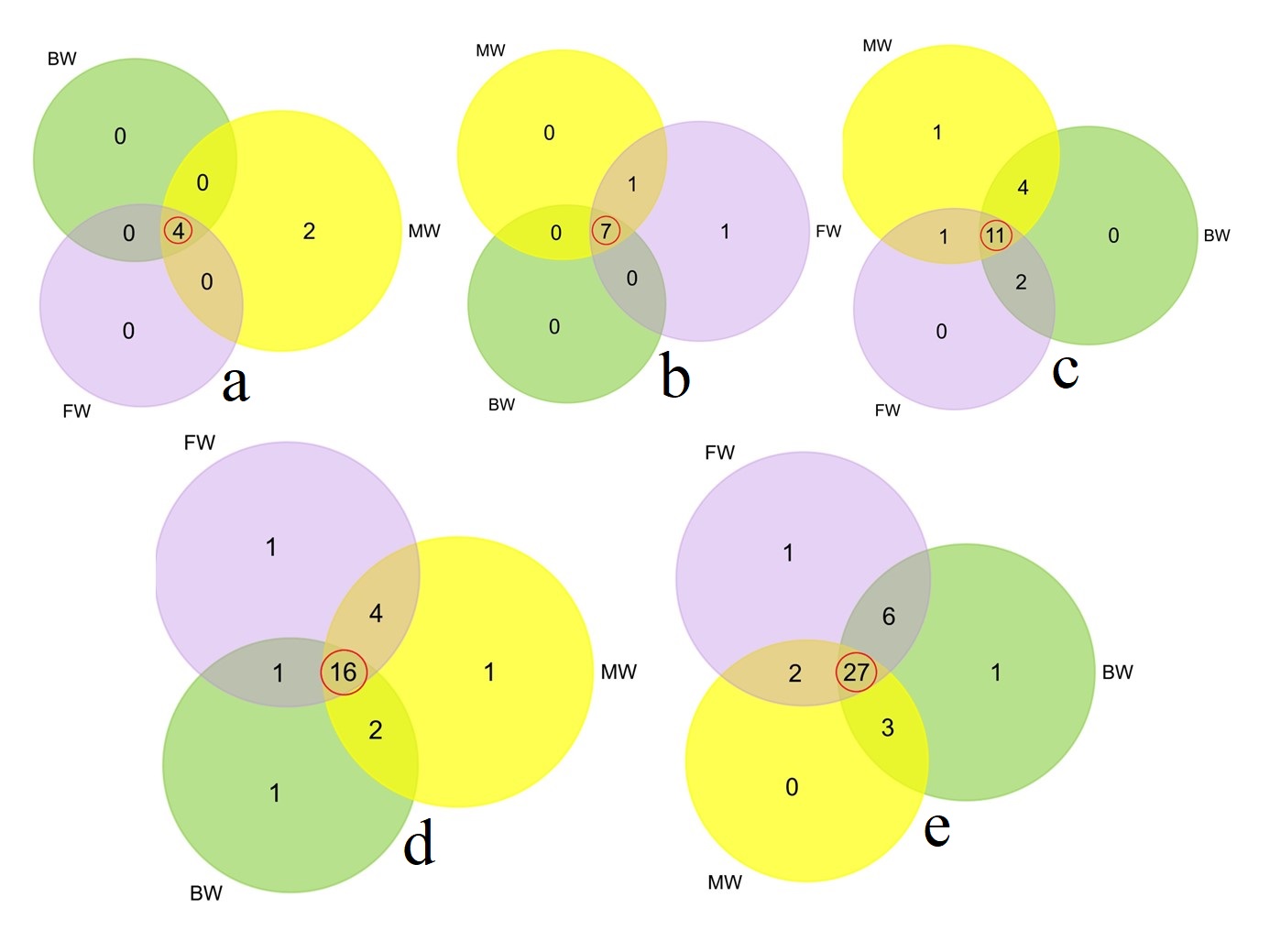


**Fig. S2. Taxonomic composition of bacteriomes**. Venn diagrams representing the unique and shared bacterial (a) phylum, (b) class, (c) order, (d) family, and (e) genus identified in the gut of hilsa fishes in three habitats e.g., freshwater (FW), brackish water (BW) and marine water (MW) of Bangladesh. Shared genera (highlighted in red circles) were found to be shared across the habitats.


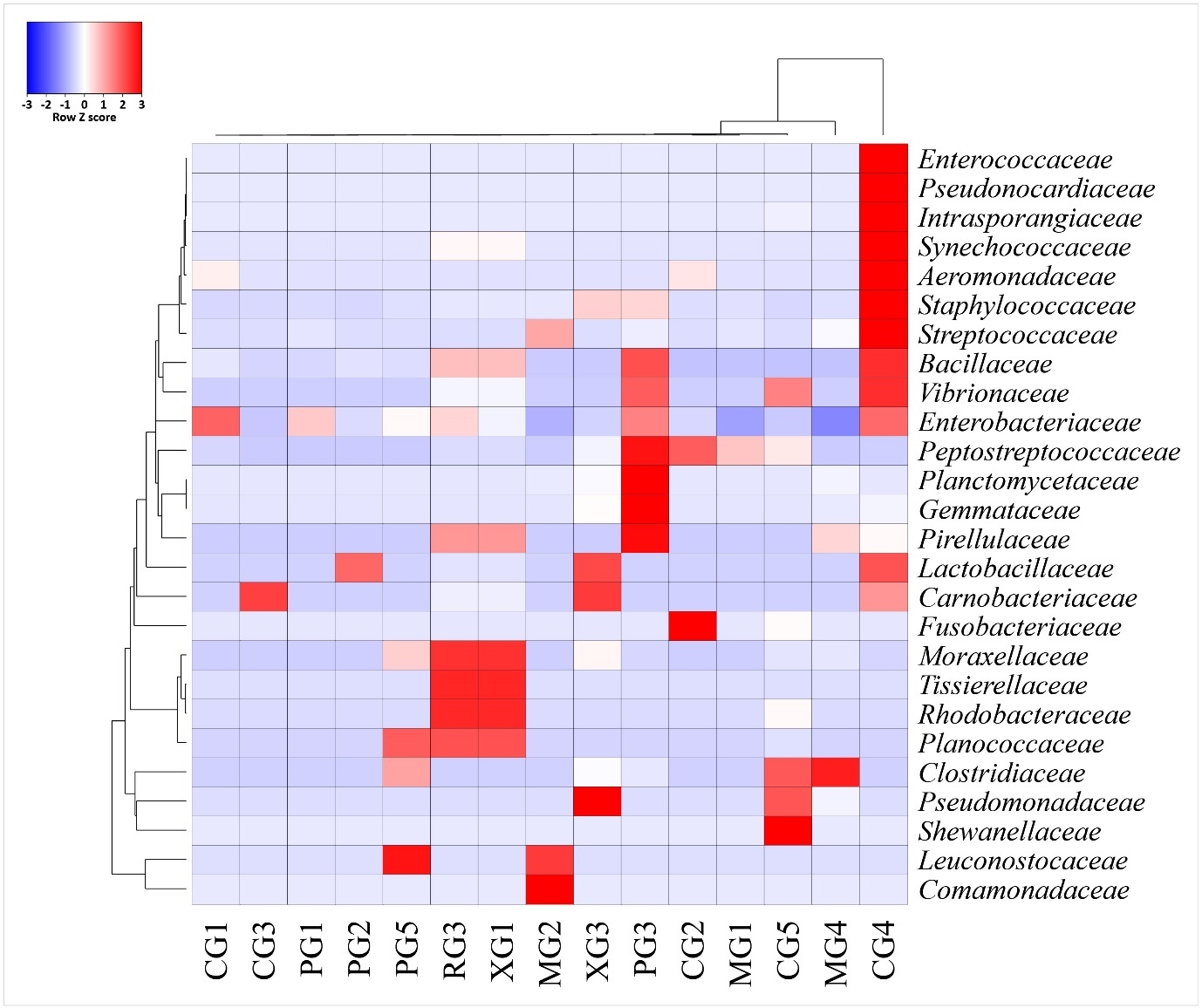


**Fig. S3. Taxonomic profile of bacteriomes in hilsa fishes at the family level.** Heatmap showing the average relative abundances and hierarchical clustering of the bacterial families in the study samples. The colour bar (row Z score) at the top represents the relative abundance of the bacterial families in the corresponding samples. The colour codes indicate the presence and completeness of each bacterial taxa, expressed as a value between =3 (lowest abundance) and 3 (highest abundance). The red colour indicates the more abundant patterns, while blue cells account for less abundant families in that particular sample.


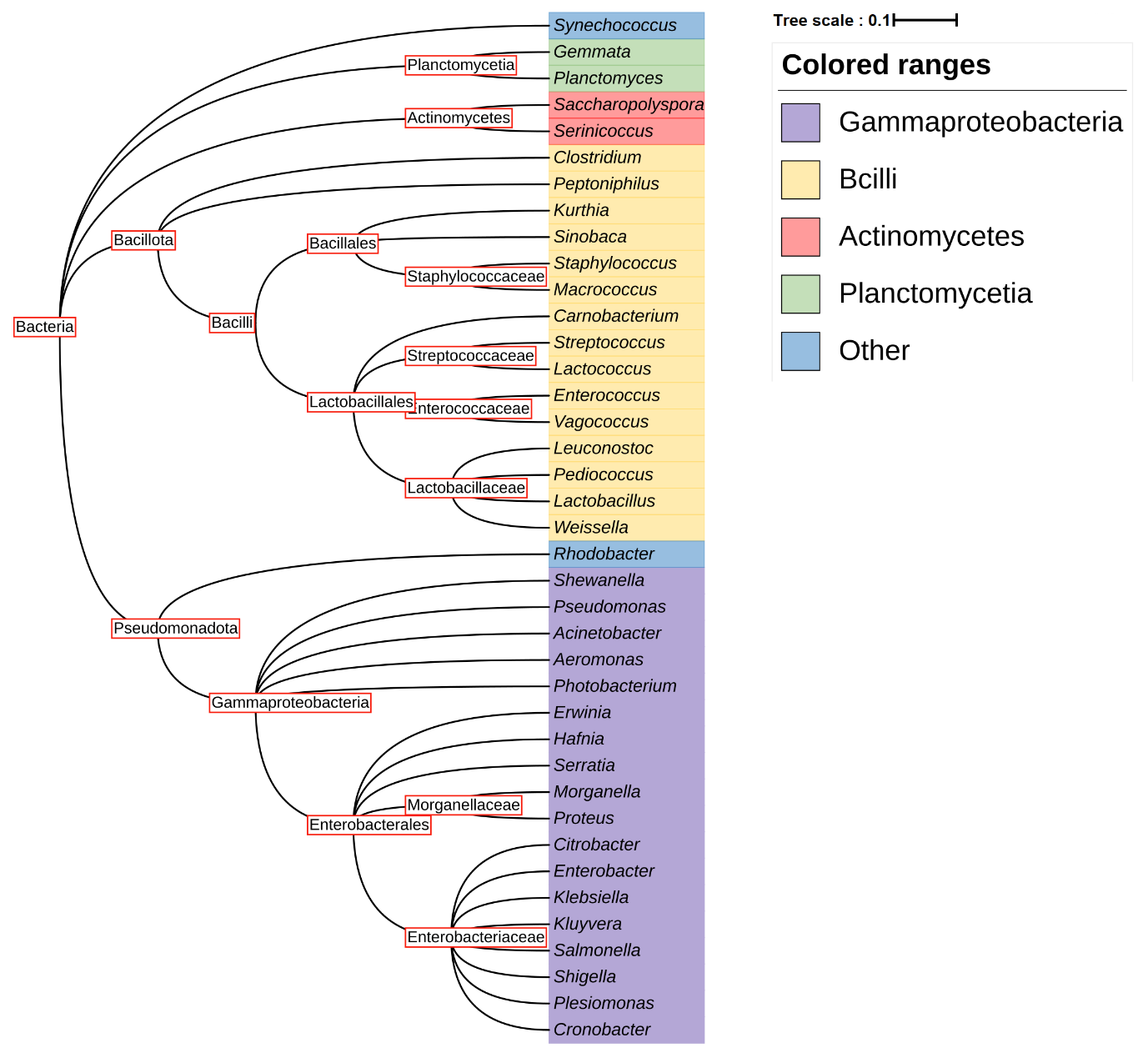


**Fig. S4. Phylogenetic relationship of the bacteriomes.** The rooted phylogenetic tree was generated with 40 genera of bacteria in the gut and flesh samples of the hilsa fishes across three habitats based on the maximum likelihood method using the NCBI taxonomy tree and visualized with iTOL (interactive Tree Of Life). The bootstrap considered 1000 replicates. The scale bar represents the expected number of substitutions averaged over all the analysed sites. The length of the scale bar represents 1 nucleotide substitution per 100 positions. Different colours are assigned according to the order level taxonomic ranks of the bacteria.

**Table S1:** Study sample information, SRA accession numbers of the 16S rRNA amplicon sequences and OTUs (operational taxonomic units) mapped against bacterial taxa.

| **Sample ID** | **Collection site** | **Coordinate** | **Habitat** | **Source** | **No. of raw reads** | **No. of mapped reads** | **No. of observed OTUs** | **SRA accessions** |
| --- | --- | --- | --- | --- | --- | --- | --- | --- |
| CG1 | Confluence of Meghna and Padma River, Chandpur | 23.2321° N, 90.6631° E | Freshwater | Gut | 325,812 | 33,295 | 22 | SRR24402593 |
| CG2 | Bay of Bengal, Cox's Bazar | 21.4272° N, 92.0058° E | Marine water | Gut | 140,124 | 12,418 | 22 | SRR24402592 |
| CG3 | Bay of Bengal, Cox's Bazar | 21.4272° N, 92.0058° E | Marine water | Gut | 119,676 | 4,738 | 10 | SRR24402608 |
| CG4 | Bay of Bengal, Cox's Bazar | 21.4272° N, 92.0058° E | Marine water | Gut | 697,544 | 291,731 | 23 | SRR24402607 |
| CG5 | Payra River, Patuakhali | 22.3586° N, 90.3317° E | Brackish water | Gut | 131,276 | 9,514 | 20 | SRR24402606 |
| RG3 | Padma River, Rajshahi | 24.3745° N, 88.6042° E | Freshwater | Gut | 194,464 | 2,256 | 18 | SRR24402605 |
| MG1 | Meghna River, Munshiganj | 23.5422° N, 90.5305° E | Freshwater | Gut | 165,096 | 1,333 | 15 | SRR24402604 |
| MG2 | Meghna River, Munshiganj | 23.5422° N, 90.5305° E | Freshwater | Gut | 126,908 | 7,353 | 15 | SRR24402602 |
| MG4 | Meghna River, Munshiganj | 23.5422° N, 90.5305° E | Freshwater | Gut | 260,420 | 3,077 | 11 | SRR24402601 |
| PG1 | Payra River, Patuakhali | 22.3586° N, 90.3317° E | Brackish water | Gut | 149,988 | 611 | 23 | SRR24402600 |
| PG2 | Payra River, Patuakhali | 22.3586° N, 90.3317° E | Brackish water | Gut | 119,052 | 1,142 | 11 | SRR24402598 |
| PG3 | Payra River, Patuakhali | 22.3586° N, 90.3317° E | Brackish water | Gut | 361,312 | 8,111 | 20 | SRR24402599 |
| PG5 | Payra River, Patuakhali | 22.3586° N, 90.3317° E | Brackish water | Gut | 178,372 | 3,982 | 18 | SRR24402597 |
| XG1 | Bay of Bengal, Cox's Bazar | 21.4272° N, 92.0058° E | Marine water | Gut | 112,684 | 509 | 16 | SRR24402596 |
| XG3 | Bay of Bengal, Cox's Bazar | 21.4272° N, 92.0058° E | Marine water | Gut | 151,480 | 260 | 14 | SRR24402595 |

**Table S2. Hilsa Samples metadata**

| Hilsa Samples | Length (cm) | Girth (cm) | Weight (g) |
| --- | --- | --- | --- |
| XG1 | 42.21 | 25.33 | 877.1 |
| XG3 | 38.53 | 24.1 | 695.2 |
| PG1 | 41.1 | 23.54 | 722.25 |
| PG2 | 41.7 | 27.27 | 916.29 |
| PG3 | 43.51 | 26.31 | 905.71 |
| PG5 | 39.11 | 24.23 | 701.42 |
| MG1 | 37.21 | 22.15 | 658.29 |
| MG2 | 40.35 | 27.1 | 880.57 |
| MG4 | 36.57 | 20.21 | 620.51 |
| CG1 | 45.12 | 29.4 | 1053.58 |
| CG2 | 43.57 | 28.71 | 1017.36 |
| CG3 | 43 | 29 | 1124.41 |
| CG4 | 43.61 | 29.53 | 1125.75 |
| CG5 | 42.14 | 29.33 | 1055.6 |
| RG3 | 18.68 | 9.9 | 56.87 |
| Average | 39.77 | 25.07 | 827.40 |

| Taxa | Freshwater | Brackish water | Marine water |
| --- | --- | --- | --- |
| Phylum (n=6) | 4 | 4 | 6 |
| Class (n=9) | 9 | 7 | 8 |
| Order (n=19) | 14 | 17 | 17 |
| Family (n=26) | 22 | 20 | 23 |
| Genus (n=40) | 36 | 37 | 32 |

**Table S3**: Taxonomic information on hilsa bacteriomes.
