# Supplementary material for: Unveiling the gut bacteriome diversity and distribution in the national fish hilsa (Tenualosa ilisha) of Bangladesh
Source: PLoS One. 2024 May 1;19(5):e0303047. doi: 10.1371/journal.pone.0303047 (PMC11062526; doi:10.1371/journal.pone.0303047)
Supplement: Attachment — Submitted filename: Responses to Reviewers.pdf [file pone.0303047.s004.pdf]

Point-by-point responses to the reviewer comments

Editor's comment

Comment 1: We noticed you have some minor occurrence of overlapping text with the following previous publication(s), which needs to be addressed: https://doi.org/10.1007/s11033-023-08965-6. In your revision ensure you cite all your sources (including your own works), and quote or rephrase any duplicated text outside the methods section. Further consideration is dependent on these concerns being addressed.

Our Response: We sincerely thank the Editor for the encouraging comments and valuable suggestions for the improvement of the manuscript. We have addressed minor text overlapping issue with a previous publication and cited that article accordingly (Biswas et al., 2024; https://doi.org/10.1007/s11033-023-08965-6). A key finding in this study was the discovery of multiple beneficial and/or probiotic bacterial genera within the gut of hilsa fish across the three distinct habitats. Indeed, the isolation and efficacy of probiotic bacteria derived from hilsa fish have not been previously documented or reported. Additionally, a noteworthy discovery from this study was the identification of certain bacterial genera (such as *Sinobaca*, *Synechococcus*, *Gemmata*, *Serinicoccus*, *Saccharopolyspora*, and *Paulinella*) that have not been reported in any aquatic or marine fish species. You may kindly go through the revised manuscript.

Comment 2: We note that the grant information you provided in the 'Funding Information' and 'Financial Disclosure' sections do not match. When you resubmit, please ensure that you provide the correct grant numbers for the awards you received for your study in the 'Funding Information' section.

Our Response: We sincerely thank you for this valuable suggestion. We have addressed this discrimination in the revised manuscript and revised submission. You may kindly go through the revised manuscript and on-line submission portal.

Comment 3: Please include a separate caption for each figure in your manuscript.

Our Response: We would like to thank the handling editor for this suggestion. We have provided a separate caption for each figure in the revised manuscript. You may kindly go through the revised manuscript.

Comment 4: Please include captions for your Supporting Information files at the end of your manuscript, and update any in-text citations to match accordingly.

Our Response: We do appreciate your suggestion. We've included captions for your Supporting Information files at the end of our manuscript, and updated in-text citations accordingly. You may kindly go through the revised manuscript.

Comment 5: We are unable to open your Supporting Information file [File Name]. Please kindly revise as necessary and re-upload.

Our Response: We apologize for your inconvenience. Thank you very much for this important suggestion. We've added all Supporting Information in a separate PDF file as well as at the end of our manuscript. You may kindly go through the revised manuscript.

Comment 6: Please review your reference list to ensure that it is complete and correct.

Our Response: Thank you very much for this concern. We've revised the reference section according to PLOS ONE format. You may kindly go through the revised manuscript.

Reviewer # 1

Reviewer comment: The manuscript entitled, Unveiling the gut bacteriome diversity and signature of the Bangladesh national fish hilsa (*Tenualosa ilisha*) (Manuscript Number: PONE-D-24-07704) by Kawser and co-worker is well written in the manuscript. However, minor modifications are required. Please see the comments.

Our Response: We sincerely thank the reviewer for the encouraging comments and valuable suggestions for the improvement of the manuscript. We would like to take this opportunity to express our sincere thanks to the expert reviewer who identified several areas in our manuscript

that were needed corrections as well as modifications. You may kindly go through the revised manuscript.

Reviewer comment: Key Words are to be Keywords.

Our Response: Thank you. We agree with your important suggestion. We've revised and updated the Keywords. You may kindly see the Keywords in the revised manuscript.

.

Reviewer comment: The abstract section is well written

Our Response: We sincerely appreciate the reviewer for such an encouraging comment.

Reviewer comment: Line no 147: used instead of utilized

Our Response: We sincerely appreciate the reviewer for these valuable suggestions. We have replaced utilized by used in the mention line. You may kindly go through **Lines 122 and 152** in the revised manuscript.

Reviewer comment: The result section is well written

Our Response: We sincerely appreciate the reviewer for such an encouraging comment.

Reviewer comment: Discussion should be more discussed with the latest literature

Our Response: We sincerely appreciate the reviewer for this valuable suggestion. We've updated the discussion section citing updated references. You may kindly go the revised manuscript.

Reviewer comment: Image quality should to improved.

Our Response: We thank the reviewer for this suggestion. This is something we are unable to address since all the images are so far in good quality and have already passed PLOS ONE publication criteria. I think the judicious reviewer will consider this issue.

Reviewer ## 2

Reviewer comment: The manuscript titled "Unveiling the gut bacteriome diversity and signature of the Bangladesh national fish hilsa (Tenualosa ilisha)" Manuscript Number: PONE-D-24-07704 is well written.

Our Response: Thank you for the encouraging comments on our work and valuable suggestions for the improvement of the manuscript.

Reviewer comment: The title should include "Diversity and distribution" of gut bacteriome.

Our Response: Thank you for this valid suggestion. We have revised the title of the manuscript according to the reviewer's suggestion. You may kindly go through the **title** in the revised manuscript.

Reviewer comment: Line No. 45 The Keywords should be *Tenualosa ilisha*, Gut microbiome, Metagenomics, 16S rRNA, Probiotic properties.

Our Response: Thank you. We agree with your important suggestion. We've revised and updated the Keywords. You may kindly see the Keywords in the revised manuscript.
